# In Vitro and In Vivo Assessment of the Potential of *Escherichia coli* Phages to Treat Infections and Survive Gastric Conditions

**DOI:** 10.3390/microorganisms9091869

**Published:** 2021-09-03

**Authors:** Joanna Kaczorowska, Eoghan Casey, Gabriele A. Lugli, Marco Ventura, David J. Clarke, Douwe van Sinderen, Jennifer Mahony

**Affiliations:** 1 School of Microbiology, University College Cork, T12 YN60 Cork, Ireland; j.m.kaczorowska@amsterdamumc.nl (J.K.); eoghan.casey@ucc.ie (E.C.); david.clarke@ucc.ie (D.J.C.); 2 APC Microbiome Ireland, University College Cork, T12 YT20 Cork, Ireland; 3 Laboratory of Probiogenomics, Department of Chemistry, Life Sciences and Environmental Sustainability, University of Parma, 43121 Parma, Italy; gabrieleandrea.lugli@unipr.it (G.A.L.); marco.ventura@unipr.it (M.V.)

**Keywords:** phage therapy, prophylactic, infections, *Galleria mellonella*, diarrheagenic *Escherichia coli*

## Abstract

Enterotoxigenic *Escherichia coli* (ETEC) and *Shigella* ssp. infections are associated with high rates of mortality, especially in infants in developing countries. Due to increasing levels of global antibiotic resistance exhibited by many pathogenic organisms, alternative strategies to combat such infections are urgently required. In this study, we evaluated the stability of five coliphages (four *Myoviridae* and one *Siphoviridae* phage) over a range of pH conditions and in simulated gastric conditions. The *Myoviridae* phages were stable across the range of pH 2 to 7, while the *Siphoviridae* phage, JK16, exhibited higher sensitivity to low pH. A composite mixture of these five phages was tested in vivo in a *Galleria mellonella* model. The obtained data clearly shows potential in treating *E. coli* infections prophylactically.

## 1. Introduction

Diarrheal diseases are among the leading causes of mortality in children under the age of five years [1,2]. Most of these deaths occur in so-called developing countries, especially sub-Saharan Africa and southern Asia [1,3]. The etiology of diarrheal diseases depends on different factors such as the age of the child and region [3]. Diarrhea is most often a symptom of an intestinal infection, caused by various species of viruses, bacteria, or parasites [2]. Two groups of bacterial pathogens, namely diarrheagenic *Escherichia coli* (DEC) and *Shigella* spp., are significantly associated with moderate-to-severe diarrhea in young children in various regions of the world [3].

DEC are currently classified into six groups: enteropathogenic *E. coli* (EPEC), enterotoxigenic *E. coli* (ETEC), enterohemorrhagic *E. coli* (EHEC), enteroinvasive *E. coli* (EIEC), enteroaggregative *E. coli* (EAEC), and diffusely adhering *E. coli* (DAEC) [4]. These pathotypes differ from each other in terms of the clinical and pathological characteristics of the infection and in the prevalence in various countries and regions [5,6,7]. In developed countries, the most common DEC infections are caused by EHEC (also referred to as verocytotoxin-producing *E. coli*, VTEC and shigatoxin-producing *E.coli*, STEC) and are usually associated with a consumption of contaminated foods [4,5]. According to the European Centre for Disease Prevention and Control (ECDC), in 2018, an excess of 8000 cases of EHEC infections were reported in the EU.

ETEC infections are much more common in developing countries and occur mainly in both children and also travelers coming from industrialized countries to less developed regions [5]. ETEC produces so-called colonization factors that enable these bacteria to adhere to small intestine mucosa [8]. *Shigella* spp. is a pathogenic enterobacteria closely related to *E. coli* [9]. *Shigella* infections are considerably more common in developing countries and, together with ETEC, are among the “top five” causative agents of children’s diarrhea in these countries [3]. This bacterial pathogen penetrates the human colonic mucosa, which leads to the disruption of the intestinal epithelium and causes both inflammation and dysentery [9].

Many *Shigella* and *E. coli* strains have become resistant to a variety of easily obtained and thus widely used antimicrobials [10,11,12]. Considering antibiotics are not recommended for the therapeutic treatment of STEC, alternative approaches such as antibody therapy, toxin receptor analogs, vaccines, and probiotic treatment strategies have been developed [13]. Furthermore, considerable efforts have been made recently to develop effective vaccinations against *Shigella* spp. and ETEC; however, the presence of a large number of epidemiologically relevant serotypes of ETEC and especially of *Shigella* spp. requires the vaccine to have a broad spectrum of protection [14,15,16,17]. A vaccine targeting many strains and conjugates is challenging to develop and is likely to be expensive, especially in the context of developing countries [14]. (Bacterio)phages are viruses that infect bacterial cells. They are very precise in targeting a host and are mostly species-specific, often even strain-specific [18]. Inter-species infection is occasionally observed, particularly among phages infecting the *Enterobacteriaceae* family, as members of this family are very closely related [18]. The targeted ability of phages to kill bacteria of certain species or genera render them as an alternative to conventional treatments of bacterial infections [18]. Conversely, the high specificity of some phages is viewed as a limitation to the therapeutic potential of phages, particularly when phages possess a narrow host range [19]. While clinical and pathogenic isolates are the preferred model for therapeutic evaluations, well-defined strain banks and collections are of considerable value also. The *E. coli* reference strain collection of 72 strains isolated from humans and 16 additional mammalian species represents a highly useful and applicable tool for the evaluation of phages in the laboratory setting. While these strains were originally considered non-pathogenic, it is known that pathogenic *E. coli* strains group among the ECOR strains based on multi-locus enzyme electrophoresis, making it a safe and representative model system for *E. coli* [20]. Furthermore, subsequent characterization of the ECOR strain collection identified that one strain, ECOR8, may be enteroaggregative, while others may be pathogenic based on the presence of virulence factors and cytotoxic activity [20].

*Galleria mellonella* (greater wax moth) larvae have been a useful model in various bacterial infection studies [21,22,23,24,25,26], including those of *Shigella* ssp. and *E. coli* [21,26]. This insect has a semi-complex innate immune response, which bears similarities to mammalian innate immunity [21]. Moreover, the larvae of *G. mellonella* tolerate a wide range of temperatures (including 37 °C) and can be easily injected or dissected [21,25]. The assessment of the presence of ill-health is also relatively easy, as the insects show evident melanization upon infection [22].

We previously established a collection of coliphages and examined both their genetic and morphological characteristics [27]. Furthermore, one of the phages (JK08) was evaluated for its potential application in a phage cocktail against a bank of clinical *E. coli* isolates in solid and liquid medium-based assays [28]. Furthermore, some of these phages have also been shown to be effective against *Shigella sonnei* 53G [27]. Certain combinations of phages were observed to cause a reduction in efficacy, possibly due to the competition for receptor binding sites. In the current study, we evaluated the pH stability of five representative coliphages from our collection, as well as their ability to withstand simulated gastric conditions. A phage cocktail incorporating five distinct phages was tested in vivo in an insect (*Galleria mellonella*) model as a therapeutic agent against single strain and multiple strain infection.

## 2. Materials and Methods

### 2.1. Bacterial Strains, Phages, and Growth Conditions

Prophage-free *Escherichia coli* strains BL21 [29], Top10 (Thermo Fischer Scientific, Waltham, MA, USA), and DH5α (NEB) were used as propagation hosts for the phages used in this study. A selection of *E. coli* strains representing the ECOR library [30], namely ECOR8, ECOR15, ECOR42, ECOR62, and ECOR70, were used in in vivo assays. For host range determination, all 72 strains of the *E.coli* reference collection (ECOR) were used [30]. Bacterial liquid cultures were grown from a single colony in LB broth (1% NaCl (Sigma Aldrich, St. Louis, MO, USA), 1% tryptone (Merck, Kenilworth, NJ, USA), and 0.5% yeast extract (Merck)) at 37 °C with aeration. All bacterial strains were preserved as glycerol stocks at −80 °C. The phages used in this study and their characteristics are presented in Table 1. Phages were propagated on the relevant host strain grown in LB broth when the cultures reached an optical density (OD_600_) of approximately 0.2 and were co-incubated at 37 °C with agitation until lysis was observed. The phage lysates were filtered twice through a 0.22 µm filter and stored at 4 °C until required for experimental assays.

### 2.2. Host Range Determination

The host range of JK16, JK36, and JK38 was previously determined against the reference *E. coli* (ECOR) strain collection of 72 strains [27,30]. The host range of JK1 and JK08 was determined against the ECOR strain collection using spot assays and confirmatory plaque assays as described previously [27]. The semi-solid agar contained 0.4% agar while the base agar contained 1% agar. Visual inspection of the plates was used to determine if zones of clearing were present after the spot assays and where such zones of clearance were observed, phage dilutions were performed in plaque assays to confirm the observation of genuine phage infection. This was performed to exclude possible false positives in the spot assays.

### 2.3. Phage DNA Isolation, Sequencing, and Analysis of the Genome of Phage JK1

Genomic DNA of phage JK1 was extracted using the Norgen Phage DNA isolation kit (Norgen Biotek, ON, Canada) according to the manufacturer’s instructions. Purified DNA was sequenced using an Illumina MiSeq Sequencing System at the GenProbio facility (Parma, Italy). Genome assemblies of the paired end reads (2 × 250 bp reads) were performed with MIRA v4.0.2, while open reading frames (ORFs) were predicted with Prodigal version 2.6. The ORFs were automatically annotated with BLAST (https://blast.ncbi.nlm.nih.gov/Blast.cgi, accessed on 6 August 2019) against NCBI and HMMER databases, while functional analysis was performed by evaluation against the web-based Pfam and HHPred databases [31,32]. The genomes were visualized and edited using Artemis Release 15.0.0 (http://www.sanger.ac.uk/science/tools/artemis, accessed on 6 August 2019) and nucleotide BLAST (BLASTn). The percentage of similarity between the phage proteins was acquired using protein BLAST (BLASTp). The genome sequence of JK1 was deposited in Genbank under the accession number MZ436830. The genome was manually evaluated for the presence of antibiotic-resistance genes and functions associated with lysogeny (repressor, superinfection immunity, and integrase) based on the outputs of the HHPred, Pfam, and BlastP analysis described above.

### 2.4. The Effect of Various pH and Simulated Gastric Fluid

The pH of the LB broth was adjusted with HCl to obtain pH values of 2, 3, 4, 5, or 6. Standard LB broth at pH 7 and was used as a control. Phage lysates with a titer of 10^9^–10^10^ PFU/mL of JK1, JK08, JK16, JK36, and JK38 were mixed with the prepared suspensions in a 1:10 ratio and incubated at 37 °C for 1 h. After incubation, the samples were transferred into SMG buffer (200 mM NaCl, 10 mM MgSO_4_ (Sigma Aldrich), 50 mM Tris-HCl (Sigma Aldrich), pH 7.5, 0.01% (*w*/*v*), and gelatin (Sigma Aldrich)). Simulated gastric fluid (SGF) was comprised of 3.2 mg/mL porcine pepsin (Sigma Aldrich) resuspended in 0.2% NaCl (*w*/*v*) at pH 2 [33]. The solution was prepared immediately prior to the experiment and filtered through a 0.2 µm membrane. Phage lysates were mixed with the prepared suspension in a 1:10 ratio and incubated at 37 °C. The samples were collected every 10 min and transferred into SMG buffer. The phage titer at “T_0_” was collected before the phage addition to the SGF and another sample, “T_2_”, was collected 2 min after transferring the phage lysate into the SGF to define the immediate impact of the SGF, if any. Subsequently, samples were taken at 10 min intervals over a period of 40 min to assess the ability of the phages to withstand the simulated gastric conditions. The phage suspensions were serial diluted in SMG buffer and the phage titers were estimated using the double layer agar method, as described previously [34], with modifications of the volumes of phage and bacterial suspensions (10 µL of diluted phage and 100 µL of overnight bacterial culture were used). The agar plates were incubated overnight at 37 °C. All assays were performed in triplicates.

### 2.5. In Vivo Phage Therapy Test Using the Galleria Mellonella Model

The in vivo experiments were performed as described previously [24], with the modifications described below.

#### 2.5.1. Bacterial Inoculum and Phage Cocktail

**Figure 1:**
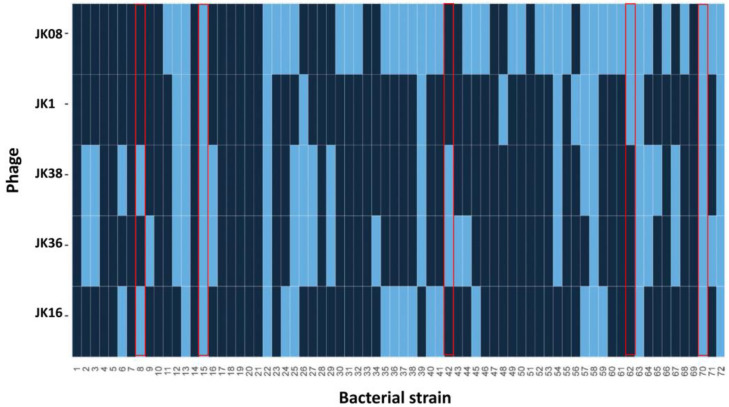
Heat map detailing the host range of phages JK1, JK08, JK16, JK36, and JK38 against the ECOR collection of 72 strains (numbered 1–72). Light blue indicates infection and dark blue indicates absence of infection. Host range data for JK16, JK36, and JK38 is adapted from [27]. Strains highlighted in a red box represent those selected for the infection model assays.

All ECOR strains were divided into three groups based on their electromorph profiles [34]. These profiles were defined based on an electrophoretic analysis of 11 enzymes important for microbial metabolism [34]. Five ECOR strains were selected for the in vivo assays: ECOR8, ECOR15, ECOR42, ECOR62, and ECOR70. Each of these strains possess different electromorph profiles within the three groups mentioned above, as follows: ECOR8 and ECOR15 are members of group I; ECOR42 belongs to group II; and ECOR62 and ECOR70 belong to group III [30]. The strains were also selected based on the host range of the phages selected for this study (Figure 1). A 1% inoculum of fresh overnight culture was added to 10 mL of LB broth and incubated at 37 °C with agitation until an OD_600_ of 0.5–0.7 was achieved. The bacterial suspension was diluted in cold LB to achieve a concentration of approximately 10^7^ CFU/mL [24] and was kept on ice during larvae injections. Phage lysates of the five selected phages were mixed to form a cocktail of approximately 10^9^ PFU/mL (final concentration) with an equal concentration of all phages present in the mixture. The multiplicity of infection (MOI) of phages relative to the number of host cells applied in the study was optimized based on in vivo trial experiments in which an MOI of approximately 1, 10, and 100 were evaluated in a single strain infection model (n = 10 for each MOI).

#### 2.5.2. Testing of Phage Cocktail against a Single Strain and against a Pool of the Strains

Larvae of *G. mellonella* were obtained from Reptile Foods Ireland (Youghal, Ireland). The insects were stored at 4 °C and used within two weeks. Larvae were selected for in vivo experiments according to their weight, which ranged between 0.2 and 0.35 g. Before the injection, larvae were surface-sterilized using cotton swabs dipped in 70% ethanol. A single dose (10 µL) of bacterial inoculum, phage cocktail, or sterile growth medium (LB) was injected into the hemolymph using a 100 µL glass Hamilton syringe. The larvae were selected randomly for each treatment regimen and incubated in a Petri dish at 37 °C. The larvae remained unfed throughout the experiment [26], which lasted a maximum of 48 h. Four control groups were specified: the first group was injected with only medium (Control LB); the second with the phage cocktail (Control PC); the third one with bacterial inoculum of a single strain (Control ECOR); and the fourth group was inoculated with a strain pool (Control strain pool). Two treatment models were tested: prophylactic and remedial. In the prophylactic treatment model, the phages were injected 2 h before the bacterial suspension (PC + ECOR/strain pool), while in the remedial treatment model, the bacteria were injected 2 and 5 h before the phages (ECOR/strain pool + PC).

## 3. Results

### 3.1. Characterization of the Phages Comprising the Cocktail

In a previous study, we screened a range of environmental and food samples for the presence of phages capable of infecting *E. coli* and/or *Shigella* [28]. The host range of the phages (including JK16, JK36, and JK38) was evaluated against the ECOR 72 reference strain collection and *Shigella sonnei* 53G, and distinct host range profiles were identified for each identified phage among the collection. In the present study, the host range of phages JK1 and JK08 was determined against the ECOR strain collection and compared to those of the previously characterized phages (JK16, JK36, and JK38). Through this analysis, it was determined that JK1 was capable of infecting 16 strains (or 22.2% of the ECOR collection), while JK08 was capable of infecting 41 strains (or 56.9%) within the strain collection (Figure 1). These phages have distinct (or overlapping) host range profiles compared to those of each other and the previously characterized JK16, JK36, and JK38. However, the combination of JK1, JK08, JK16, JK36, and JK38 ensures that approximately 80% of the ECOR collection of strains is targeted by at least one of the phages. Strains 1, 4, 5, 7, 10, 14, 17–21, 28, 33, 47, 51, 66, and 69 are not infected by any of the five phages. A readily identifiable link between these strains and the reason underpinning their resistance cannot be deduced but they may encode phage-resistance systems or not possess the appropriate receptor for the tested phages.

Recently, the International Committee on Taxonomy of Viruses (ICTV) have reclassified the Caudovirales order of tailed phages. This order previously incorporated three families of phages termed *Myoviridae, Siphoviridae*, and *Podoviridae*. This has been revised and 14 families, 73 subfamilies, 927 genera, and 2814 species are now described based on comparative genome analysis (https://talk.ictvonline.org/ictv-reports/ictv_9th_report/dsdna-viruses-2011/w/dsdna_viruses/67/caudovirales, accessed on 9 June 2021). Among these, *Siphoviridae* previously represented the cohort of phages with long non-contractile tails irrespective of genetic composition. Based on comparative genome analysis, phages with long non-contractile tails may be members of the *Siphoviridae*, *Demerecviridae*, and *Drexlerviridae* (formerly termed the T1 superfamily) families [35]. JK16 was previously characterized as a *Siphoviridae*, while under the recent reclassification scheme, it is a member of the *Warickvirus* genus of the *Drexlerviridae* family. Furthermore, *Myoviridae* and their component genera have similarly been dissected and both JK08 and JK38 represent distinct isolates of the *Tequatrovirus* genus. Additionally, JK36 is a member of the *Mosigvirus* genus of the *Myoviridae* family and bears 97% of its nucleotide identity to the well-studied phage RB69 over 93% of its genome (Table 1).

In the present study, the genome of an additional phage isolated from river water in the Republic of Ireland was sequenced and analyzed. BlastN of the whole genome sequence of JK1 revealed high sequence-relatedness (97% identity over 97% genome sequence) to the coliphage teqsoen, isolated from Danish wastewater [36], and the coliphage T2 (97% identity over 92% of the genome). Therefore, we propose that JK1 is a new member of the *Tequatrovirus* (T4-like) genus of the *Myoviridae* family. The genome of JK1 possesses 97.57% and 94.53% sequence identity over 96% and 91% of the genomes of JK38 and JK08, respectively. Both of these phages have previously been characterized as T4-like phages [27]. The region encoding the tail fiber and the tail fiber adhesin is the most notable region of divergence between the three Tequatroviruses and is consistent with their distinct (although overlapping with nine strains infected by all three phages) host range profiles (Figure 1). Furthermore, JK1 shares almost 80% of its nucleotide identity across 50% of the genome of JK36, highlighting the genetic distinction of these phages.

The genome of JK1 was observed to comprise 166,057 bp with 266 predicted open reading frames (ORFs). The genome is predicted to possess a mol percentage of GC content of 35.48, which is identical to that of JK38 [27] and slightly higher than that of JK08 (35.38%), both of which belong to the *Tequatrovirus* genus. Furthermore, the genome was analyzed for the presence of lysogeny-associated functions and virulence factors, and neither were observed, indicating that the phage possessed potential for application in phage therapy trials. Among the 266 predicted ORFs, a function could be proposed for 132 gene products and functions associated with capsid, and tail morphogenesis was readily discernible as were replication-associated functions. Within the proposed morphogenesis modules are several genes whose products are typically associated with *Myoviridae* phages. These include predicted baseplate wedge components (ORFs 148-153), short tail fiber (Gp12-like), tail sheath, and sheath stabilizer/completion proteins. Based on previous studies of T-even *Myoviridae*, it is predicted that the Gp38-like adhesin encoded by ORF242 and the Gp12-like short tail fiber encoded by ORF155 are likely the major contributors to host range determination. BlastP analysis of the short tail fiber protein identified that this protein exhibits >95% aa identity to Gp12 proteins of coliphages T2 and T6, as well as 93% aa identity to that of T4. Furthermore, it presents 100% sequence identity to the equivalent in JK38; however, it exhibited reduced sequence identity to those of JK36 (81.9%) and JK08 (65%). The strong overlap of the host range (13/16 strains infected by JK1) of JK1 and JK38 corroborates the function and role of the Gp12-like short tail fiber in the host range determination of these phages. Similarly, the Gp38 adhesin of JK1 and JK38 are 100% identical while no significant similarity to that of JK36 and only 48% aa identity (55% query coverage) to that of JK08. This suggests that JK1 and JK38 are highly likely to recognize and bind to similar receptors on their hosts’ cell surface, distinct from those of the other Myophages JK08 and JK36.

**Figure 2:**
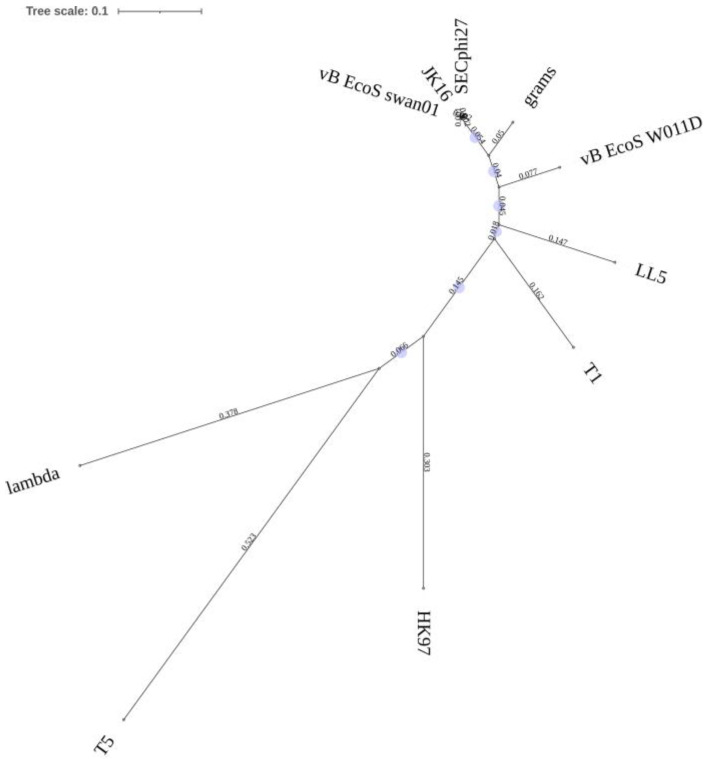
Unrooted phylogenetic tree of the receptor binding proteins (RBPs) encoded by *Escherichia coli* phages. The JK16 RBP is most closely related to those of SECphi27 and vB_EcoS_swan01 while it is distinct from those of phages belonging to distant phage genera.

JK16 is the sole phage in the tested cohort to present with a non-contractile tail. The predicted receptor-binding protein (RBP) of this phage is encoded by ORF75. HHpred analysis of this protein (1192 aa) identified an oligosaccharide-binding/baseplate domain (PDB entry 6TEH_D) with 99.5% probability (amino acids 307-489). The BlastP analysis of the proposed RBP identified its sequence similarity to the RBPs of other Warwickvirus members (including *Escherichia* phages vB_EcoS_swan01 and SECphi27; >99% aa identity over 100% of the protein sequence) and a reduced sequence similarity to those of other genera within the Tempevirinae sub-family, such as grams (89% identity; 100% query coverage), vB_EcoS-W011D (78% identity; 100% query coverage), and LL5 (70% identity; 100% query coverage). Sequence alignments of the RBP sequences revealed that the C-termini is the most divergent region of the protein sequences and is consistent with the typical host-specific binding domains of RBPs. Furthermore, the RBP of JK16 shares limited sequence-relatedness to those of phages belonging to distant *Escherichia* phage genera (of non-contractile tailed phages), including Byrnievirus HK97, Lambdavirus lambda, and Tequintavirus T5 (Figure 2). The RBP of T5, termed the L-shaped fiber, binds reversibly to polymannose O-antigen domains of the *E. coli* LPS [37].

Based on these genomes and host range data, five representative phages, i.e., JK1, JK08, JK16, JK36, and JK38, were selected to evaluate their efficacy in in vitro and in vivo trials.

### 3.2. Evaluation of O-Antigens as Phage Receptors

Several coliphages have been described to bind reversibly to (poly)saccharide components [37,38,39] and often comprise a component of the lipopolysaccharide (LPS). Many strains within the ECOR collection present the O-antigen [40], which is a cell surface LPS moiety that acts as a receptor for certain *E. coli* and *Salmonella* phages [41]. The LPS of the outer membrane of Gram-negative bacteria may be of the rough (R) or smooth (S) form, the latter of which is associated with the presence of the O-antigen [42]. The genomes of the ECOR strain collection have been sequenced and analyzed; however, it is difficult to define precisely which strains harbor the O-antigen due to the presence of contaminating sequences and multiple O and/or H molecular serotyping loci [43,44]. Four of the five strains are predicted to produce a R1 core polysaccharide (PS; strains ECOR 15, 42, 62, and 70), while one is predicted to produce a R2 core PS structure (strain ECOR 8) [40] to which the antigenic PS side-chain is attached. Ribotyping of the ECOR collection allowed for the discernment of O and H antigen types of its component strains. Among these, the five strains selected for application in this study (ECOR 8, 15, 42, 62, and 70) are believed to produce distinct O-antigens (O86, O25, ON, O2, and O78, respectively, and where ON refers to an untypable antigen using standard antisera) [45]. The high variability of the O-antigen side-chain structures likely contributes to the specificity of tailed phages that employ this reversible binding step. It is noteworthy that certain phages, including T5, may “bypass” this reversible binding step and bind directly to a proteinaceous receptor if the O-antigen receptor is absent [37,46]. Considering each of the five phages selected in this study have overlapping host ranges, it is unlikely that the O-antigen is the primary/sole determinant of the observed host range of the phages applied in this study. Interestingly, several strains including ECOR 13, 15, 22, 58, 63, 70, and 72 are infected by all five phages evaluated in this study (Figure 1). This suggests that the phages either recognize a common receptor that is present in all of these strains or that the strains present multiple receptor moieties facilitating infection by a number of different phages. Three of these strains (ECOR 13, 22, and 63) produce O-antigens that are untyped using standard anti-sera, while ECOR 15, 58, 70, and 72 produce distinct O-antigens (O25, O112, O78, and O144, respectively) [45]. Therefore, while the initial and reversible binding step may be performed via distinct O-antigen (or other) moieties, it appears that the irreversible receptor is the more significant determinant of the host range.

### 3.3. Stability Is Highly Phage-Specific

**Figure 3:**
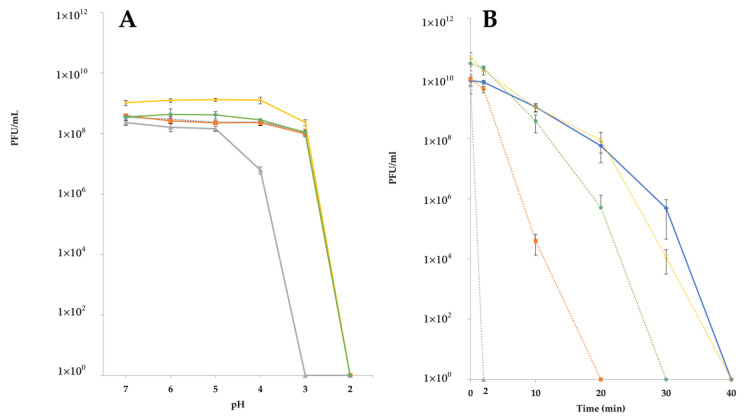
(**A**) Phage survival across a range of pH values: the graph shows the phage counts of each of the tested phages after 1 h incubation at 37 °C and at specific pH values. (**B**) Phage survival in SGF, pH 2, over time; the graph shows the phage counts of each of the tested phages after 0, 2, 10, 20, 30, and 40 min of incubation in SGF. Each colored line represents a distinct phage: blue = JK1; orange = JK08; grey = JK16; yellow = JK36; and green = JK38. All assays were performed in triplicates and the graphs represent the average and standard deviation error bars of these data.

Oral delivery of (phage-containing) medicines seems to be the easiest and most convenient among all other possible delivery routes. The main obstacle for this kind of delivery method is the phage stability in low pH and proteolytic environment of the gastrointestinal tract [47]. The five phages tested in this study were evaluated for their stability in low pH conditions (Figure 3). All phages remained stable at pH 5, 6, and 7, and all except JK16 were shown to retain their infectivity at pH 4, with no significant impact on phage titer (Figure 3A). JK16 exhibited a two-log reduction in efficiency of plaquing at pH 4 and at pH 3 it was undetectable (Figure 3A). JK1, JK08, JK36, and JK38 showed less than a log reduction in titer at pH 3. However, at pH 2, there were no active phage particles detected (Figure 3A). It is noteworthy that JK16 is the sole *Drexlerviridae* representative [28] and displays the greatest sensitivity to low pH treatment, while the *Myoviridae* representatives behaved similarly and appeared to elicit a higher robustness in the trials. Each of the phages were also tested for their ability to survive in SGF. The experiment was performed over a period of 40 min, with phage titer evaluations performed every 10 min. Of all tested phages, JK1 and JK36 were shown to retain the highest level of infectivity for the longest exposure duration in SGF (Figure 3B). There were still 10^4^ PFU/mL and 10^5^ PFU/mL of infective phage particles of JK1 and JK36, respectively, after 30 min of incubation (Figure 3B). Conversely, phages JK38 and JK08 were completely inactivated after 30 min and 20 min, respectively (Figure 3B). Phage JK16, which exhibited high susceptibility to low pH, was fully inactivated 2 min after its transfer into the SGF (“T_2_”sample; *p*-value < 0.05) (Figure 3B). One-way ANOVA analysis of the survival data at each time-point for each of the remaining four phages relative to the average starting titer showed a statistically significant *p*-value (<0.05) for phages JK1/JK36 relative to JK08 and JK38 at the 10 to 40 min time-points (Figure 3B).

### 3.4. In Vivo Testing Reveals the Potential of Prophylactic Treatment of Multi-Strain E. coli Infections

**Figure 4:**
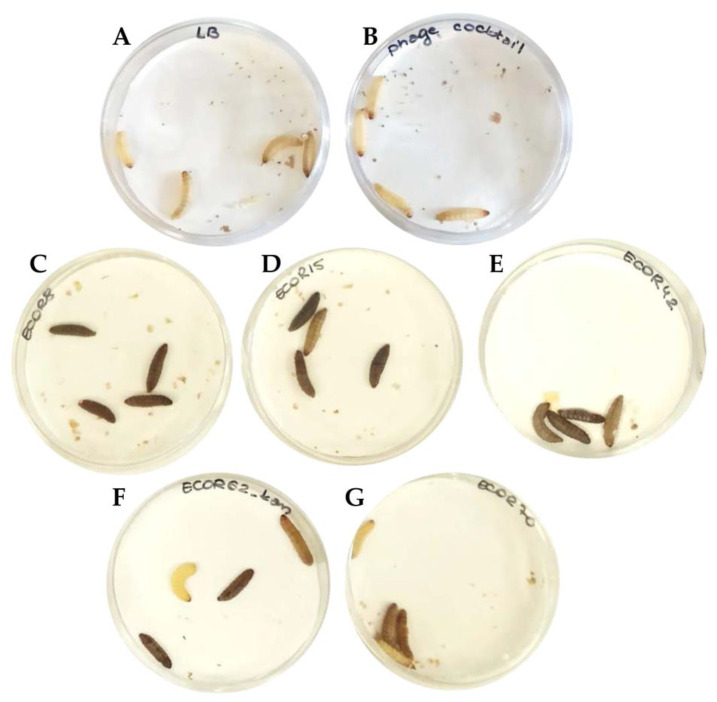
Evaluation of the impact of ECOR strains on *Galleria mellonella* melanization and/or survival. Controls of the background medium LB (**A**) and the phage cocktail (**B**) were evaluated. Larvae were injected with suspensions containing approximately 10^5^ cells of individual ECOR strains, namely ECOR8 (**C**), ECOR15 (**D**), ECOR42 (**E**), ECOR62 (**F**), and ECOR70 (**G**). The degree of melanization was recorded after 16 h incubation at 37 °C.

As mentioned above, the phage lysates included in the cocktail were propagated on prophage-free and non-pathogenic laboratory strains. Moreover, the sequences of the phages were known to be free of any lysogeny-related genes or gene-encoding toxins, which may have an influence on the in vivo assay [28,29]. Nevertheless, it was important to assess if either the phage cocktail or the growth media (LB) had any influence on *G. mellonella* larvae survival. Four larvae were injected with 10 µL of LB (Control LB) and another four with a freshly pooled phage cocktail (Control PC) (Figure 4A,B). All the larvae from both control regimens remained healthy and yellow colored with no traces of melanization until the end of experiment (Figure 4A,B). All strains were tested for the ability to cause infection in the larvae and in all cases, the larvae showed melanization 16 h post-injection. The level of melanization varied among different strains (Figure 4C–G). Infection with ECOR70 caused a less intense melanization than the remainder of the assessed strains (Figure 4G). The concentration of bacterial cells required to cause melanization in all tested larvae (*n* = 10) w evaluated using two strains, ECOR62 and ECOR72, and in both cases, 10^5^ cells was the optimal concentration (below this concentration, melanization was observed in less than 50% of the larvae tested). ECOR 72 was not used in further trials; however, the cell concentration defined using this preliminary analysis was applied to all strains in all subsequent single and multi-strain infection models.

**Figure 5:**
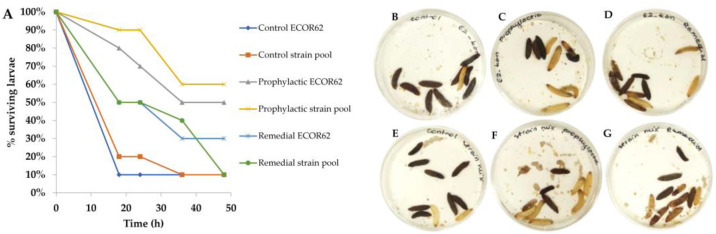
Evaluation of prophylactic and remedial phage therapy treatments of larvae infected with a single strain (ECOR62) or a mixture of five ECOR strains (*n* = 10). The number of surviving larvae after 48 h incubation at 37 °C was recorded and plotted (**A**). Controls in which the single strain ECOR62 alone (**B**) or strain combinations (**E**) were included. Prophylactic (**C**,**F**) and remedial (**D**,**G**) treatments of the single or combined strain pool, respectively, at an MOI of 100, were evaluated for their therapeutic potential. Prophylactic treatment was observed to be most effective in both single and mixed-strain assays. The number of larvae displaying melanization after the incubation period was recorded and plotted in the graph. Similar effects of the individual strain and strain pool were observed, while the larvae group treated with phages prophylactically displayed higher survivability and reduced melanization compared to the control and remedially treated cohorts. Panels (**B**–**G**) represent the results at the 16 h time-point.

To ascertain the efficacy of phages in treating *E. coli* infections in vivo, we compared a single strain infection with a multiple strain infection model. In this experiment, we evaluated two phage cocktail treatments—remedial and prophylactic—using a time window of 2 and 5 h. We used the phage cocktail comprised of approximately equal amounts of each phage and tested both treatments in a cohort of 10 larvae. The multiplicity of infection (MOI) differed slightly depending on the bacterial strain and phage. Optimization trials with a single strain infection model with ECOR62 established that an MOI of 1 or 10 provided negligible (0–20%) protection and that an MOI of 100 was required to allow for survival of the larvae in prophylactic treatment models (100%). ECOR62 and subsequent single strain infection trials were selected for this, as it is infected by only two phages from the cocktail, representing a limited infection range scenario. To maintain the same testing conditions (and extent of dilution/competition between the phages), all five phages were included in both the single and multi-strain infection models. A graph displaying changes in the larvae survival over time is presented in Figure 5A. The bacterial control groups, i.e., in the treatment groups injected only with bacteria, the percentage of live larvae dropped by 80% to 90% after 16 h (Figure 4 and Figure 5A,B,E), with just 10% survival in both control groups by the experimental end-point (Figure 5A,B,E). The prophylactic treatment of single strain infections resulted in a 40% increase of larvae survival relative to the untreated control, as five (of ten) larvae remained alive and healthy 48 h post-infection (Figure 5A,C). The prophylactic treatment of the multi-strain infection allowed six (out of ten) larvae to survive and remain healthy until the end of the experiment (Figure 5A,F). The remedial treatment of infection with single ECOR strain resulted in just 30% larvae survival (Figure 5A,D). In the case of multiple strain infection treated remedially, 40% of the larvae survived 36 h post-infection, with a sudden noticeable drop in the survival in the last time-point (Figure 5A,G).

## 4. Discussion

Alternative and inexpensive methods to treat intestinal bacterial infections in the developing world are urgently needed [11,48]. Phages seem to be well-suited for applications in the developing countries: they are readily isolated and can be prepared both relatively easily and cheaply, and many have been shown to retain activity in the powder form, notwithstanding the cost associated with production under GMP (good manufacturing practice) conditions [48,49,50,51]. As the easiest route of drug administration to the intestine is the oral route, the phages within the cocktail should be resistant to the low pH of the stomach [47]. In the current study, we tested the stability of selected coliphages across a range of pH values and in simulated gastric conditions. The efficacy of a phage cocktail against single and multi-strain *E. coli* infections was also determined. Through this analysis, a highly phage-specific reaction to low pH and simulated gastric conditions was observed, highlighting the need for detailed characterization of individual phages destined for therapeutic trials. Interestingly, the *Drexlerviridae* phage JK16 appeared highly susceptible to both low pH and SGF, suggesting its low suitability for oral administration. Low pH survival is a routinely evaluated feature of phages with therapeutic potential [52,53,54,55,56]. Jurczak-Kurek and colleagues indicated that all isolates within their collection of 83 phages were fully deactivated after 1 h incubation at pH 2 [52]. Moreover, most of the phages showed a significant decrease in titers at pH 4 [52], which is consistent with the outcome for *Drexlervirus* JK16 in the present study (Figure 1). Interestingly, those phages displaying 100% retention of infectivity at pH 4 in the Jurczak-Kurek study were classified as *Myoviridae*, while no *Siphoviridae* (phages with long non-contractile tails) phage exhibited complete survival at this pH [52]. Therefore, we speculate that *Myoviridae* phages may present greater applicability to phage cocktails that are to be delivered orally.

Given the limitations of the oral delivery of certain phages, encapsulation of the phage particles may provide a means of delivery to the intestinal site. Microencapsulation of the Felix O1 *Salmonella* phage into chitosan-alginate capsules [32] resulted in a significantly higher infectivity retention of the phage at low pH and in bile salts in comparison to the free phage, and the phages were released in the pH conditions of the porcine gut [32].

The phage cocktail studied in this paper reflects promising potential to treat *E. coli* infections in vivo. Most importantly, the control treatment with only the phage cocktail did not cause any visible symptoms (in this case, melanization) in *G. mellonella* larvae and, on this basis, we consider that either endotoxins are absent from the lysates used in this study or that they are at very low levels (Figure 4). This observation suggests that the studied phage cocktail is most probably safe and free of any toxins or contaminants. As for the phage cocktail treatments of bacterial infections, we observed a considerable increase in the survival of larvae treated with the phage cocktail prophylactically compared to the bacterial controls (Figure 5). The survival rate was similar for both the single strain infection and multi-strain infection model (Figure 5). In the present study, the dose of bacterial cells administered was based on an initial evaluation of the dose required to cause melanization in all the tested larvae (*n* = 10) for two strains, namely ECOR62 and ECOR72. However, the lethal dose of each bacterial strain may differ. Therefore, while the dose of 10^5^ cfu/mL was selected as the dose for all the bacterial strains in this study, based on preliminary evidence from two strains, the dose for each strain should be determined and preferably should apply clinical isolates that are relevant to the specific phage/phage cocktail under evaluation if it is to be applied therapeutically. The remedial treatment was unsuccessful in the case of the multi-strain infection and had lower efficacy in single strain infection models compared to the prophylactic treatment (Figure 5). The advantage of the prophylactic treatment over the remedial treatment in the insect model was described previously in the work by Nale et al., which focused on phage cocktails against *Clostridium difficile* [23]. To evaluate the genuine potential of phages, such as those applied in this study, it would be valuable to ascertain if the phages can be recovered after the test period, which would define the survivability of the phages in vivo. This would also allow for a comparison of the survivability in simulated gastric conditions (as per Figure 3) and in the gastrointestinal tract. Furthermore, the pH of the larval gastrointestinal tract could also be established to define the likely survivability of the phages. To date, there are no examples of published results exploring phage therapy against *E. coli* or *Shigella* spp. in the *Galleria mellonella* model; however, other animal models testing the efficacy of *E. coli* phage cocktails have been reported; for example, there have been mouse models with varying success [19]. Furthermore, human trials exploring the potential and safety of phage therapy against *E. coli* and *Shigella* spp. are needed. The T4 phage cocktail was implemented in a clinical trial in Bangladesh with no adverse effects associated with the phage treatments observed [57]. Prophylactic treatment of dysentery in humans has been successfully implemented in Georgia [58]. In this case, the therapy was applied to children during the peak months of *Shigella* spp. infections [58]. The application of the phage cocktail decreased the incidence of dysentery by 3.8 folds [58]. However, unsuccessful clinical trials of phage treatments have also been reported. For example, in the work of Sarker et al., the orally distributed phages failed to improve the health of children suffering from EHEC-associated diarrhea, even though the phages survived the gastrointestinal passage [59]. Clearly, it is important to explore the remedial therapy potential of *E. coli* phage cocktails and further work should be undertaken to improve these treatments. For example, in a previously mentioned study by Nale et al., a combined therapy (phage cocktail + antibiotic) was studied and proved to increase the survival rate of *G. mellonella* larvae infected with *C. difficile* [24], which thus could be trialed for other organisms in the future. In the present study, an MOI of 100 was required for effective treatment. While this may be achievable for many *E. coli* phages and possibly other Gram-negative phages, it is noteworthy that such MOIs may be difficult to achieve in some species as some phages do not propagate to high titers under laboratory conditions. This attribute is an important consideration for a phage destined for therapeutic applications.

## 5. Conclusions

In the present study, we evaluated the ability of five distinct phages to survive simulated gastric conditions (low pH and in the presence of bile). Furthermore, we aimed to establish if their combined presence would have an impact on the prophylactic or remedial treatment of single or multi-strain infections (competitive exclusion based on receptor saturation, for example). The five phages, which comprised our phage cocktail, showed varying levels of susceptibility to acidic pH and survival in SGF. The insect model demonstrated that the tested phage cocktail exhibits potential in treating *E. coli* infections prophylactically. The *G. mellonella* model proved to be a useful model for the preliminary investigation of the efficacy of these phages, although it is not without its limitations. Therefore, future studies should focus on an extensive evaluation of the optimal MOI of phages to apply and investigate alternative model systems or cell lines, while the possible synergy between phages and probiotics/vaccines in treating *Shigella* and *E. coli* infections should also be evaluated thoroughly. Additionally, prior to large-scale testing of such phage cocktails against single or multi-strain infections, the concentration of the cells of each strain should be determined to identify strain-specific effects. Many unknown factors remain in the application of phage therapy; however, it is imperative that continuing studies evaluating the potential of such treatments are performed. The presence of endotoxins that may shed from host cells and present in phage lysates through the propagation process should be evaluated to discern their genuine application potential. It is also important that phages are evaluated using similar tools and methods to allow for comparisons between the efficacy of different phage isolates. Ultimately, additional models using higher organisms will be required to evaluate the potential of such phages to treat human infections. Furthermore, the observation of the higher efficacy of prophylactic treatment requires further investigation and should be a consideration for other similar phage evaluation studies.

## Figures and Tables

**Table 1:** Characteristics of the phages applied in this study.

Phage Name	Source	Propagation Host	Family	Genus	Reference
JK1	River Lee, Cork	BL21	*Myoviridae*	*Tequatrovirus*	This study
JK08	Cork City stream	DH5α	*Myoviridae*	*Tequatrovirus*	[29]
JK16	Cork City stream	DH5α	*Drexlerviridae*	*Warickvirus*	[28]
JK36	Sewage	Top10	*Myoviridae*	*Mosigvirus*	[28]
JK38	Sewage	BL21	*Myoviridae*	*Tequatrovirus*	[28]

## Data Availability

The JK1 genome sequence was deposited in the Genbank database under the accession number MZ436830.
